# Study of NBT–Pluronic F–127 Gels as 1D UV Radiation Dosimeters for Measurement of Artificial Light Sources

**DOI:** 10.3390/ma15072370

**Published:** 2022-03-23

**Authors:** Elżbieta Sąsiadek-Andrzejczak, Agata Mądrakowska, Marek Kozicki

**Affiliations:** Department of Mechanical Engineering, Informatics and Chemistry of Polymer Materials, Lodz University of Technology, Żeromskiego 116, 90-543 Lodz, Poland; 219049@edu.p.lodz.pl

**Keywords:** UV radiation, radiochromic hydrogels, nitro blue tetrazolium chloride (NBT), Pluronic F–127, 1D radiation measurements, UV radiation dosimeters, artificial UV radiation sources

## Abstract

This work reports on radiochromic dosimeters for 1D UV light measurements. The dosimeter is composed of a 25% Pluronic F–127 that forms a physical gel matrix and nitro blue tetrazolium chloride (NBT) as a radiation-sensitive compound. This dosimeter was exposed to UVA, UVB and UVC radiation, and the radiochromic reactions were followed with reflectance spectrophotometry including changes in light reflectance and color coordinates in the CIELAB color system. The exposition of dosimeters to all UV radiation caused color changes from pale yellow to dark violet, and its intensity increased with increasing absorbed dose. The effects of NBT concentration and UV radiation type on the dose–response of the dosimeters were also examined. The results obtained reveal that the dosimeters are the least sensitive to irradiation with UVC and the most sensitive to irradiation with UVB (e.g., dosimeter with 2 g/dm^3^ of NBT was characterized by the following parameters: the threshold dose 0.1 J/cm^2^; the dose sensitivity −5.97 ± 0.69 cm^2^/J; the linear dose range 0.1–2.5 J/cm^2^; the dynamic dose range was equal to 0.1–3 J/cm^2^). The results obtained reveal that the NBT–Pluronic F–127 dosimeters can be potentially useful as 1D sensors for artificial UV radiation sources measurements.

## 1. Introduction

UV radiation has a positive effect on human health. Absorption of ultraviolet type A (UVA) and type B (UVB) radiation by the skin depends on the phototype, which is determined on the Fitzpatrick scale [1] and defines the tendency relative to burns and the risk of skin cancer. Insufficient exposure of the body to UVA and UVB radiation reduces the production of vitamin D3, which lowers the resistance of the immune system, increases cancer incidence, causes hypertension and cardiovascular disease and also causes multiple sclerosis, Alzheimer’s disease, autism, asthma, type 1 diabetes and myopia [2,3]. However, the excess of UV radiation may have a detrimental effect on the body and cause, e.g., DNA damage, immune disorders, eye damage and tumors [4,5,6,7]. 

Regarding artificial UV light sources, some studies have reported using them to treat people with parchment skin and have found that internal light sources such as fluorescent lamps, quartz halogen and tungsten filament bulbs emit UVA, UVB and even UVC radiation. Moreover, the intensity of UV radiation from some artificial sources is similar to that of the sun. Therefore, commonly used internal light sources can pose a health risk, especially for people with medical conditions that make them sensitive to light [8]. However, according to the International Commission on Non-Ionizing Radiation Protection (ICNIRP) and the World Health Organization (WHO), there are no recommendations for dosimeters for monitoring UV radiation generated by artificial light sources.

UV radiation is also a harmful factor for employees from various professional groups, e.g., welders, medical workers or nail stylists [9]. Despite the massive popularity of cosmetic procedures, there is still not enough information about the hazards related to the use of UV cabinets for curing gel, hybrid and titanium nails in beauty salons and at home. In addition, UV radiation is necessary for the proper performance of the service, because the composition of gels and varnishes includes dyes, glitters, metallized threads, foils, sequins, oligomers, monomers and photoinitiators. UV cabinets commonly used for this purpose are usually equipped with fluorescent bulbs or light-emitting diodes (LEDs). The emission spectrum of fluorescent lamps is from 300 nm to 410 nm, with a maximum emission of 375 nm. The LED lamps emit UV radiation in the range from 375 nm to 425 nm with the highest emission value at 385 nm. Therefore, in both cases, radiation from the UVA and UVB ranges is emitted [10]. Research shows that the nail plate completely blocks UVB radiation and allows the penetration of only 0.5–2.5% of UVA radiation [11]. However, the skin of the fingers and the back of the hand is exposed to UV radiation, which can absorb a dose equal to annual exposure during one manicure treatment. Despite such studies, it is widely believed that the use of UV lamps for nails does not significantly increase the risk of skin cancer, but it is strongly recommended to use sunscreen or protective gloves during exposure [10].

The use of Sun Protection Factor (SPF) filters and protective clothing, including gloves, is one of the recommended methods of UV protection for workers from various professions. The proper application of SPF on the skin requires the use of 2 mg/cm^2^ sunscreen cream, which guarantees an optimal level of protection against erythema [12]. However, studies have shown that users apply a smaller amount of sunscreen cream than the recommended amount for which measurements are made, thus achieving 20–50% of the marked SPF value. In SPF filters, photostability is also a valid factor that determines the lack of loss of protective properties under the influence of UV radiation, heat and time. In the commercial market, there are two types of SPF filters (physical and chemical). The physical filters are minerals in the form of micro-powders of zinc oxide and titanium dioxide, and their actions are based on the reflection and scattering of UV radiation. The advantage of physical filters is a very high photoprotection coefficient. Such filters penetrate the skin, absorb UV radiation and turn it into thermal energy, but they are not resistant to abrasion and must be applied additionally. Chemical filters are activated when they penetrate the skin and should be applied about half an hour before exposure to UV radiation. Due to low photostability, it is also necessary to reapply them every 2–3 h. They are highly recommended for individuals with sensitive skin, allergies and young children. Contrary to physical filters, they leave a white glow on the skin, which many users consider to be a disadvantage of such protective products [13].

Textile products used as protective clothing against UV radiation must have appropriate structural parameters such as thickness, porosity, extensibility, chemical and physicochemical properties of fibres, reflectance, finishing treatment, humidity or the presence of ultraviolet absorbers [14]. For measuring the protection of textiles against UV radiation, the ultraviolet protection factor (UPF) is used. UPF determines the ratio of the dose of solar radiation causing erythema on textile protected skin to the dose of solar radiation causing the same effect on skin without any protection. For the measurement of exposure to UV radiation of natural origin, the UV Index is used, which is a number that is linearly related to the intensity of UV radiation, causing sunburn at a given point on the Earth’s surface. The UV Index value includes the height of the sun, time of day, year, latitude and altitude, stratospheric ozone level, cloudiness and reflection of UV rays from the ground [12]. However, this method does not apply to artificial sources of UV radiation. To determine the radiation dose from artificial UV sources needed to induce erythema on the skin, a measurement of Standard Erythema Dose (SED) is used. Thus, it was determined that the value of 1 SED corresponds to exposure to UV radiation at a dose of 100 J/m^2^ [15]. Moreover, the maximum annual exposure should not exceed an erythemal-weighted dose of 15 kJ/m^2^ (150 SED) for white-skinned people [9]. Therefore, various measurement methods related to the assessment of harmful radiation doses are used, e.g., electronic meters and photodiodes [16], photodiodes and actinometers [17], sensors based on inorganic materials [18], solutions of photoluminescent dyes and liquid crystal mixtures [19,20] or biological dosimeters [21]. In addition to such solutions, dosimeters with radiation-sensitive compounds that change color under exposure to UV radiation can also be used, e.g., 1D polymer tablets [22] and 2D flat polymer films doped with tetrazolium salts [23], as well as 2D flexible flat textile modified with gels on the surface [24,25,26] or printed [27,28] and doped with 10,12–pentacosadiyonic acid polyacrylonitrile fibres [29,30]. 

Thus far, radiochromic hydrogels with Pluronic F–127 have been studied as 1D, 2D and 3D dosimeters for UV and ionizing radiation dose measurements, such as (i) textile dosimeters printed with NBT–Pluronic F–127 pastes [31]; (ii) LCV–Pluronic F–127 [32,33]; (iii) TTC–Pluronic F–127; and (iv) LMG–Pluronic F–127 [34,35]. UV dosimeters developed so far are described with the following dose–response characteristics. The TTC–Pluronic F–127 3D dosimeters were the most sensitive to UVA radiation, whereas the LMG–Pluronic F–127 3D dosimeters were the most sensitive to UVB. The dose sensitivity of the dosimeters amounted to 3.60 ± 0.12 and 42.85 ± 1.53 cm^2^/J, respectively (the linear dose range was equal to 0.15–0.9 and 0–0.3 J/cm^2^, respectively; the dynamic dose range was equal to 0–2 and 0–5 J/cm^2^, respectively). Moreover, for the 3D TTC–Pluronic F–127 dosimeters, the dose distribution is permanent over time after irradiation, whereas it was slowly lost over time for LMG–Pluronic F–127. The LCV–Pluronic F–127 2D dosimeters were also interesting, as they showed the highest sensitivity to UVB radiation and they were characterized by the following parameters: The threshold dose was below 0.005 J/cm^2^; the dose sensitivity amounted to −1.32 ± 0.11 cm^2^/J; the linear dose range was equal to 0.005–0.025 J/cm^2^; the dynamic dose range was equal to 0–3 J/cm^2^ [33]. Moreover, following former research, NBT–Pluronic F–127 dosimeters were examined for 3D radiotherapy ionising radiation dosimetry purposes and 2D textile printed dosimeters for UV radiation measurements. The studies showed that textiles printed with NBT–Pluronic F–127 pastes were the most sensitive to UVB. The NBT–Pluronic F–127 paste with 2 g/dm^3^ of NBT was characterized by the following parameters: The threshold dose was 0.01 J/cm^2^; the dose sensitivity amounted to 0.0719 ± 0.0016 cm^2^/J; the linear dose range was equal to 0–3 J/cm^2^; the dynamic dose range was equal to 0.01–3 J/cm^2^ [33]. However, NBT-Pluronic F–127 has not been characterized as a 1D gel dosimeter for UV measurements from artificial radiation sources with reflectance spectrophotometry readout. 

It was assumed that 1D dosimeters for the measurement of UV radiation from artificial radiation sources must comply with the following requirements: (i) the dosimeter must have a transparent gel matrix with low light scattering, which is related to the correct reading of the dosimeter; (ii) radio-sensitive ingredient infusing a gel matrix must respond to the UVA, UVB and UVC radiation; (iii) response to the UV radiation by color transformation of the radiation-sensitive ingredient; (iv) dosimeters are stable before and after irradiation. Therefore, the scope of work included: (i), based on previous research with radiochromic dosimeters, a polymer matrix and a compound sensitive to UV radiation were chosen; a decision was made regarding the potential use of NBT-Pluronic F–127 for 1D UV dosimetry; (ii) checking the UV radiation transmittance of the containers in which dosimeters were prepared; (iii) measurements of the light reflectance of the dosimeters before and after irradiation; (iv) calibration of dosimeters after irradiation of UVA, UVB and UVC radiation; (v) assessment of the basic parameters characterizing the dosimetry system; (vi) measurements of dosimeters stability before and after irradiation; and (vii) assessment of color changes in the CIELAB color system. In this work, we present another possibility of using such radiochromic hydrogels and a new approach for the preparation of 1D dosimeters for monitoring UV doses generated by artificial light sources. For this aim, the NBT–Pluronic F–127 radiochromic hydrogel was selected. We report NBT–Pluronic F–127 composition characteristics relative to UVA, UVB and UVC irradiation. A method of 1D dose measurement is shown with a reflectance spectrophotometer as a readout. Finally, a discussion on the application as well as weak and strong features of NBT–Pluronic F–127 for UV radiation measurements from artificial sources is included in this work.

## 2. Materials and Methods

### 2.1. Preparation of Samples

Radiochromic samples were composed of distilled water, poly(ethylene oxide)–*block*–poly(propylene oxide)–*block*–poly(ethylene oxide) (Pluronic F–127, Sigma-Aldrich, Saint Louis, MO, USA) as a gel matrix and nitro blue tetrazolium chloride (M = 817.64 g/mol) (NBT, Roth, Karlsruhe, Germany) as a radiation-sensitive compound. The use of Pluronic F–127, provides a colorless and transparent hydrogel matrix that does not change color after UV irradiation [34,35,36]. Moreover, Pluronic F–127 is approved by the US Food and Drug Administration (FDA) as a nontoxic copolymer [34]. Therefore, the developed dosimeter is not harmful to the environment and can be disposed of. Depending on ambient temperature and concentration, Pluronic F–127 can form an aqueous solution or physical gel. For instance, a 25% Pluronic F–127 solution is in the form of a physical gel in the temperature range of 19–85 °C [37], and at 5 °C, it is a solution that is easily mixed with an aqueous solution of a UV-sensitive compound.

The NBT was dissolved in distilled water at room temperature using a magnetic stirrer. Then, the solution was placed in a refrigerator (5 °C; 15 min) and then mixed with cooled Pluronic F–127 aqueous solution (33% *w*/*w*) prepared 72 h earlier. The final concentration of Pluronic F–127 in the hydrogel was equal to 25% *w*/*w* and the concentration of NBT was 1, 2 and 5 g/dm^3^. The procedure for preparing the Pluronic F–127 pre-solution is described elsewhere [34]. Afterwards, the solution was poured into poly(methyl methacrylate) (PMMA, Roth, Karlsruhe, Germany) round plastic containers with caps (outside diameter: 37 mm; inside diameter: 33 mm; the thickness of the container (height): 7 mm; the height of the gel inside the container: ~3 mm; the volume of gel solution was equal to about 2.5 mL). It is known that the preparation method can alter the reflectance of light with respect to the samples. Gel parameters such as thickness or number of layers may influence the dose–response of the dosimeter. The structure of the materials may change the absorption (K) and scattering (S) coefficients (Kubelka–Munk theory, K/S = [(1 − R_∞_)^2^/2 R_∞_], where R_∞_ is the reflectance of a sample of infinite thickness to light of a given wavelength, expressed in fractional form [27]), which are known to other materials [38,39,40]. Therefore, all samples were prepared in the same manner. No other methods of sample preparation were used, i.e., gels of different thicknesses, multilayer gels, etc. Thus, the influence of such structures on the response of the dosimeter to the UV dose was not investigated. The shape and dimensions of the containers allowed the manufacture of circular, flat dosimeters that could be easily measured with a reflectance spectrophotometer. In addition, the PMMA containers reduced the drying out of the gel. Then, the samples were covered with aluminium foil to avoid accidental exposure to daylight and stored at room temperature (23 °C). After 15 min, the sol–gel transition occurred. The samples were irradiated (Section 2.2) and measured with a reflectance spectrophotometer (Section 2.3). 

### 2.2. Irradiation of Samples

All radiochromic samples (gels in PMMA containers) were irradiated in UV-curing cabinets (UVP, Toronto, ON, Canada) at three wavelengths corresponding to UVA (8 W, type F8T5 Blacklight, range 315–400 nm; a peak at 369 nm, Hitachi, Tokyo, Japan), UVB (8 W, type G8T5E, range: 280–360 nm; a peak at 306 nm, Sankyo Denki, Tokyo, Japan) and UVC (8 W, type G8T5, 253.7 nm, Sankyo Denki, Tokyo, Japan). Each cabinet was equipped with five UV lamps. A given UV dose (J/cm^2^) was delivered automatically using a built-in detector and the control system of the device. The samples were irradiated with UVA, UVB, and UVC in a dose range of 0–3 J/cm^2^.

Some samples were also irradiated without plastic caps to assess the dose–response effect of the NBT–Pluronic F–127 dosimeter and to determine if PMMA is absorbing UV radiation. Based on the obtained results and similar studies (Figure 4 from [33]), it was found that PMMA caps absorb the most UVC and only a slight amount of UVB and UVA radiation. Thus, the actual dose delivered to NBT–Pluronic F–127 dosimeter samples covered with PMMA caps is lower by 10, 12 and 18% for UVA, UVB and UVC, respectively (the dose (J/cm^2^) indicated below in the figures is regarded as the dose emitted by the UV irradiators). Since the caps absorb UVC radiation to a great extent, the dose–response for NBT–Pluronic F–127 is hindered. 

### 2.3. Reflectance Spectrophotometry Measurements

The reflectance spectra of the NBT–Pluronic F–127 samples were measured using a light reflectance instrument (Spectraflash 300, D65/10; 10 nm resolution, the measurement error is 0.1%, DataColor, Rotkreuz, Switzerland). UV light was automatically cut off by the software so as not to irradiate the samples during the measurements, which would falsify the results. The samples were measured immediately after irradiation and over time after irradiation (for 7 days), over the wavelength range of 400–700 nm. The stability of dosimeters has been studied over a longer period (21 days). However, after approximately 8 days, air bubbles appeared in the NBT–Pluronic F–127 gel structure (drying of the hydrogel). Therefore, the obtained results of the light reflectance measurement were uncertain and were not presented in this study. Afterwards, the wavelength for which the change of the reflectance was maximal was selected and discussed vs. absorbed UV dose. Based on reflectance measurements, the characteristic parameters of NBT–Pluronic dosimeters were determined: dose sensitivity, linear and dynamic dose–response and threshold dose. Additionally, the color coordinates were determined by the CIE Lab evaluation system, which describes the perceived color according to ISO/CIE 11664-4 standard [41].

### 2.4. Stability of Samples

The stability of samples was also assessed over time before and after UV exposition. The measurements were made for the samples at the temperature of 23 °C. During the experiment, the samples were covered with Parafilm^®^ and an aluminium foil to limit the drying of the hydrogel and the access of light to the samples. The in-time stability measurements were made for non-irradiated and UV irradiated samples after 1, 2, 3 and 7 days after preparation. 

## 3. Results and Discussion

### 3.1. NBT–Pluronic F–127 UV-Dose Response

**Figure 1:**
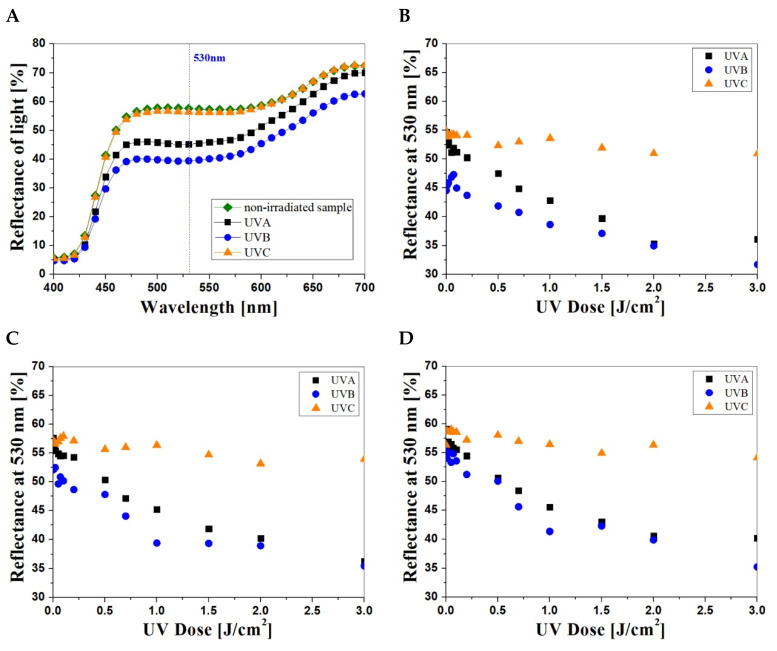
Example reflectance spectra of the NBT–Pluronic F–127 (2 g/dm^3^ of NBT) samples irradiated to 1 J/cm^2^ with UVA, UVB and UVC (**A**). The dose–responses of NBT–Pluronic F–127 hydrogels containing 1 g/dm^3^ (**B**), 2 g/dm^3^ (**C**) and 5 g/dm^3^ (**D**) NBT irradiated with UVA, UVB and UVC radiation in the dose range of 0–3 J/cm^2^.

Samples of NBT–Pluronic F–127, prepared as described in Section 2.1, were irradiated in the range of 0–3 J/cm^2^ in UVA, UVB and UVC cabinets. Afterward, they were examined with a reflectance spectrophotometer. Based on the analysis of the reflectance spectra (Figure 1A), the wavelength of 530 nm was selected. For this wavelength, the maximal changes in the reflectance of the irradiated samples were observed. Figure 1B–D show the reflectance of the NBT–Pluronic F–127 samples (NBT concentration: 1 g/dm^3^, Figure 1B; 2 g/dm^3^, Figure 1C; and 5 g/dm^3^, Figure 1D) after irradiation with UVA, UVB and UVC.

Regardless of the NBT concentration, the dosimeter samples showed visible color change, from light yellow to purple, after UVA and UVB irradiation (Table 1). No color change was observed for the samples irradiated with UVC. In this case, the samples irradiated in the dose range of 0.01–0.5 J/cm^2^ appeared to be almost the same in color regardless of the radiation dose. The measurements difference of light reflectance at 530 nm for non-irradiated and irradiated with 10 J/cm^2^ of UVC samples is approximately 4% for samples with 1, 2 g/dm^3^ of NBT and 2% for 5 g/dm^3^ of NBT. The intensity of color of the samples containing 2 and 5 g/dm^3^ NBT is slightly higher after UVC irradiation above 1 J/cm^2^. This is different from the effect obtained after irradiation with UVA and UVB, for which the intense color appeared at lower doses. Moreover, the purple color characteristic of NBT formazan formed after irradiation is shifted towards brown, which results from the intense yellow color of NBT solutions of higher concentrations; the NBT–Pluronic F–127 samples have a distinct yellow color immediately after preparation. A comparison of the L, a and b values from the CIE system of single caps after irradiation with UVA, UVB and UVC radiation in the dose range of 0–3 J/cm^2^ is presented in Table 1. The results prove that the color intensity of the irradiated NBT–Pluronic F–127 samples increases with the increase in the dose of UV radiation. The color intensity changes are the most visible to the naked eye for samples with 5 g/dm^3^ NBT. 

Based on the above measurements, the characteristic features of NBT–Pluronic F–127 dosimeters were also determined (Table 2), such as threshold dose (R_0_), which is the minimum dose of radiation needed to cause a visible change of the light reflectance spectrum of the sample; dynamic dose response range, which is the response of the system to the dose until saturation (plateau of the calibration curve), linear dose range and dose sensitivity of the system, which is the slope of the linear regression. The obtained parameters of the dosimeters confirm the previous visual observations of color changes after UV irradiation.

Comparing the values of the measuring ranges and the sensitivity, it was found that NBT–Pluronic F–127 dosimeters containing 5 g/dm^3^ of NBT are suitable for UVA radiation measurements in the dose range of 0.1–3 J/cm^2^, and their sensitivity is higher than that of the dosimeters containing 1 and 2 g/dm^3^ NBT by 14% and 60%, respectively. Dosimeters with 1 and 2 g/dm^3^ of NBT responded similarly to all types of UV radiation.

### 3.2. Stability of Samples

**Figure 2:**
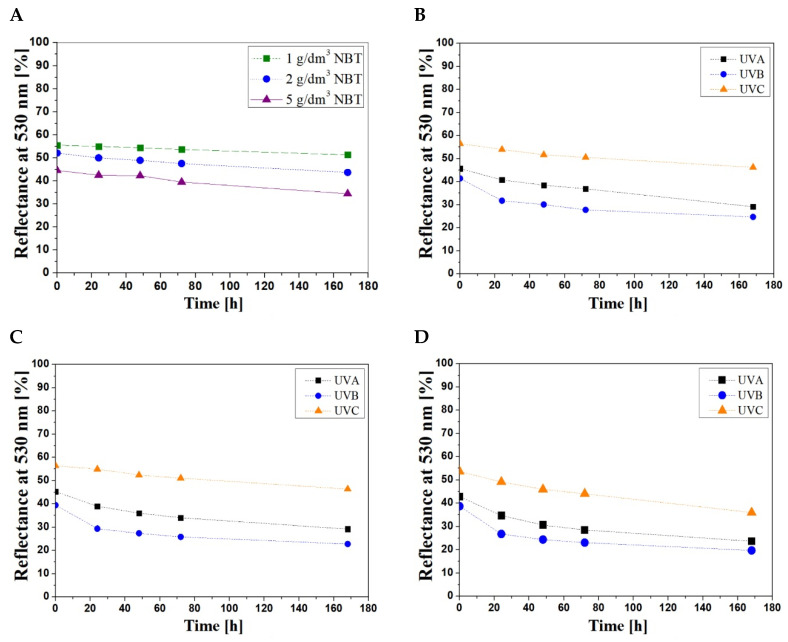
The stability of non-irradiated NBT–Pluronic F–127 samples (**A**) and irradiated samples (1 J/cm^2^) of various NBT concentrations: 1 g/dm^3^ (**B**), 2 g/dm^3^ (**C**) and 5 g/dm^3^ (**D**).

It was observed that the samples of NBT–Pluronic F–127 were not stable over time of storage despite carefully covering them with an aluminium foil between the reflectance measurements. Therefore, the reflectance of the samples containing different concentrations of NBT both non-irradiated and irradiated with UVA, UVB and UVC (1 J/cm^2^) was measured over the time of storage (Figure 2). It can be seen that the increase in NBT concentration reduces the stability of the dosimeter samples during storage. The color change of the non-irradiated samples is due to the spontaneous transformation of NBT into formazan. This study did not cover the optimization of the dosimeter’s stability, which might be conducted by the adaptation of pH or the addition of UV retarders to lower the spontaneous transformation of NBT–Pluronic F–127 dosimeters. Both, however, might impact on the dose-response performance of the dosimeter.

The reflectance measurements showed that the color change of the non-irradiated samples within 7 days was equal to 8, 16 and 22% for concentrations 1, 2 and 5 g/dm^3^ NBT, respectively. Based on the obtained data, it was found that the dosimeter containing 1 g/dm^3^ NBT seems to be the most stable over the time of storage. To determine the stability of the samples after UV irradiation, the results for one dose (1 J/cm^2^) for the samples of each NBT concentration were selected and characterized for 0–7 days of storage (Figure 2B–D). The percent change in light reflectance at 530 nm is presented in Table 3. 

Regardless of the NBT concentration, the smallest changes in the stability were observed for the samples irradiated with UVC, for which the changes in light reflectance were equal to 18, 32 and 33% for the concentrations 1, 2 and 5 g/dm^3^ NBT, respectively. The samples of NBT–Pluronic F–127 irradiated with UVB were the most unstable over time. For these samples, the change in the value of the light reflectance after 24 h was equal to 23, 30 and 31%, respectively, for the above-mentioned concentrations of NBT. The most unstable are samples containing 5 g/dm^3^ NBT. In this case, the reflectance changes (after 24 h) include 44, 49 and 33% after UVA, UVB and UVC exposition, respectively.

### 3.3. Proposition of Application

According to the International Commission on Non-Ionizing Radiation Protection, in cooperation with the World Health Organization (WHO), only the maximum daily dose for workers exposed to UV is determined [9]. This dose has been established at 30 J/m^2^, which corresponds to a little less than 1/3 of SED. The value takes into account the average DNA repair capacity in cells. However, harmful health effects occur from the total dose of UV radiation, which includes the intensity of radiation and the duration of exposure. Unfortunately, actual data on exposure to UV radiation from artificial radiation sources are very scarce. Thus, the need for reliable data on emissions from different types of lamps should be emphasized. According to the European Lamp Companies Federation, all artificial sources should be characterized by providing detailed information on the emission spectrum at the nanometre resolution [42]. Therefore, the manufacturers of lamps should label their products and provide information about photo-biological hazards: (i) actinic UV hazard for eye and skin; (ii) UVA-hazard for the eye; (iii) blue-light hazard for the retina; (iv) thermal retina hazard; and (v) IR-hazard for the eye. As stated by Standard EN 62471, lamps are then classified according to the Risk Group (RG): (i) exempt from risk (RG0); (ii) minor risk (RG1—lamps do not pose any hazards during normal circumstances); (iii) medium risk (RG2—lamps with very bright light do not pose hazards, can trigger thermal discomfort.); and (iv) high risk (RG3—include only lamps where a short-term exposure poses a hazard) [43]. The above classification is based on acute responses to exposure (up to 8 h) and applies only to individuals of normal sensitivity. Individual sensitivity to UV radiation depends on skin type, genetics, age and previous exposure to the sun [9,44]. For erythema, this is described by the minimal erythema dose (MED)—the dose of erythemal-weighted UV radiation that causes only perceptible erythema of the skin. For fair skin (Fitzpatrick type II), one MED is approximately two to three SEDs [45,46]. In addition, the annual exposure to UV radiation for type II skin should not exceed 150 SEDs [9]. Thus, according to the recommendations mentioned above, it was found that the developed NBT–Pluronic F–127 dosimeters are suitable as personal dosimeters for the measurements of the UV radiation doses absorbed by human skin from artificial sources of light. If SED (10 mJ/cm^2^) and MED (25 mJ/cm^2^, Fitzpatrick type II skin) are taken into account, which denotes one standard erythemal dose and one minimal erythemal dose, respectively, NBT–Pluronic F–127 may be useful for such measurements due to its dynamic dose–response range. For example, a dosimeter with 2 g/dm^3^ of NBT, can measure radiation in the range of 20–300 SED, 7–300 SED and 70–300 SED for UVA, UVB and UVC, respectively. However, due to the reported instability over time, they require further optimization. 

### 3.4. Pros and Cons of the NBT–Pluronic F–127 Dosimetric System

The limitations of elaborated the NBT–Pluronic F–127 dosimetric systems are as follows: (i) the reflectance spectrophotometer has specific measuring apertures that cannot be replaced, e.g., DataColor Spectraflash 300 spectrophotometer, and has aperture for flat materials with a diameter of 2 cm, which prevents the measurement of larger surfaces of dosimeters; the use of a smaller aperture and container is possible and would reduce the costs of dosimeter preparation; (ii) the PMMA containers have a limited transmission of UV radiation, especially in the UVC range; changing the material of the containers used for dosimeters preparation may improve the system’s response to UVC radiation; (iii) usage of PMMA containers does not guarantee full tightness of samples, as the NBT–Pluronic F–127 gel dries up over 7 days after preparation; containers made of a different material should be used or they should be additionally secured to prevent drying of the dosimeter; (iv) used PMMA containers have a limited capacity, so the effect of the thickness of the gel on the response to the UV dose cannot be tested; (v) the stability of dosimeters over time, especially after irradiation, is insufficient and should be refined in further research. However, the advantages of the developed dosimeters are as follows: (i) fast and simple preparation of the NBT–Pluronic F–127 gel; (ii) the use of plastic containers reduces the contact of the dosimeter with the external environment and protects the gel against mechanical damage and biological contamination; (iii) visible color change can be assessed by comparison with a color standard or by precise measurement with a reflectance spectrophotometer, but does not require specialized personnel; (iv) after irradiation, the dosimeter can be disposed of in an environmentally friendly manner; (v) the measuring range of the NBT–Pluronic F–127 dosimeters corresponds to the requirements for artificial UV radiation sources, especially in the range of UVA and UVB radiation.

## 4. Conclusions

The NBT–Pluronic F–127 dosimeters show a response to UVA, UVB and UVC radiation, regardless of the NBT concentration. The higher concentration of NBT, the more sensitive the dosimeter is to UV radiation. The NBT–Pluronic F–127 dosimeter in plastic closed containers responds best to UVB radiation and the least to UVC. The dosimeters, e.g., with 2 g/dm^3^ of NBT irradiated with UVB, were characterized by the following parameters: (i) the threshold dose was 0.01 J/cm^2^; (ii) the dose sensitivity amounted to −5.97 ± 0.69 cm^2^/J; (iii) the linear dose range was equal to 0.1–2.5 J/cm^2^; (iv) the dynamic dose range was equal to 0.1–3 J/cm^2^. Non-irradiated NBT–Pluronic F–127 samples show good stability over time regardless of NBT concentration, but they must be stored at room temperature and covered with a light-impermeable foil. However, the NBT–Pluronic F–127 dosimeters show low stability overtime after UV irradiation. The higher the concentration of NBT, the more noticeable the changes of light reflectance and color intensity over time during storage. The developed dosimeters can be used for UV radiation doses measurements in a wide range (e.g., dosimeter containing 2 g/dm^3^ NBT: 0.2–3 J/cm^2^ (UVA); 0.07–3 J/cm^2^ (UVB) and 0.7–3 J/cm^2^ (UVC)). Color changes of dosimeters can be compared organoleptically with a color scale without specialized tools or by determining the color coordinates in the CIELab system. In addition, it has been shown that NBT–Pluronic F–127 can record radiation doses in the range of the maximum annual value of skin exposure to UV radiation (up to 150 SEDs). It is assumed that further work should concern the modification of the chemical composition of the dosimeters to stabilize the spontaneous reaction of NBT and increase stability over time, especially after irradiation. Further application studies of the NBT–Pluronic F–127 on the possibility of using such systems for monitoring radiation doses from sunlight in the natural environment will enrich their characteristics as well.

## Figures and Tables

**Table 1:** Comparison of NBT–Pluronic F–127 samples with different concentrations of NBT irradiated with UVA, UVB and UVC.

NBT [g/dm^3^]	Dose [J/cm^2^]	UVA	L	a	b	UVB	L	a	b	UVC	L	a	b
1	0	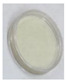	79.54	−3.86	14.78	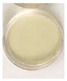	77.57	−2.64	14.92	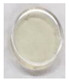	73.79	−2.06	18.84
	0.5	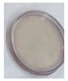	74.83	1.13	11.91	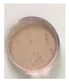	70.60	4.71	10.55	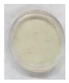	74.91	−1.76	13.06
	1	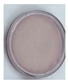	68.17	6.10	9.47	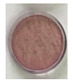	60.53	10.04	6.52	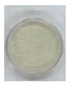	74.62	−1.25	13.00
	3	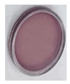	60.37	14.57	4.91	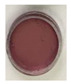	50.32	20.16	3.25	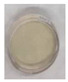	73.31	0.49	12.02
2	0	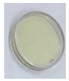	78.19	−4.58	20.98	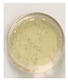	73.10	−2.27	19.33	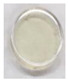	73.79	−2.06	18.84
	0.5	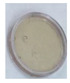	72.46	0.42	18.69	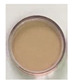	68.54	4.59	16.05	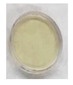	72.76	−1.32	18.44
	1	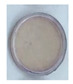	69.19	4.12	15.88	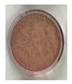	59.04	10.19	12.26	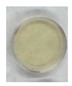	75.14	−1.28	18.79
	3	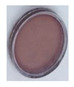	57.39	14.38	10.93	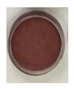	48.60	20.09	6.43	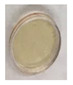	72.18	0.37	18.06
5	0	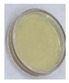	75.2	−4.02	31.14	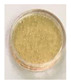	67.19	−0.19	27.67	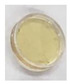	69.00	−0.22	27.77
	0.5	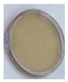	68.09	1.9	27.90	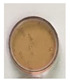	63.24	6.50	24.09	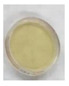	67.93	−0.02	25.59
	1	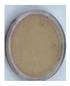	65.09	5.09	25.78	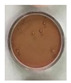	56.90	13.03	20.97	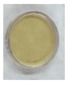	68.56	0.39	25.29
	3	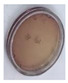	60.55	9.01	21.46	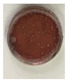	43.62	20.41	11.81	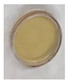	68.5	2.41	26.70

**Table 2:** Basic characteristics of NBT–Pluronic F–127 dosimeters.

NBT (g/dm^3^)	UV Type	Threshold Dose R_0_ (J/cm^2^)	Measuring Range (J/cm^2^)	Range of Linear Dose–Response (J/cm^2^)	Sensitivity A (cm^2^/J)	A_0_ Intercept	R^2^
1	UVA	0.2	0.2–3.00	0.2–2.5	−8.23 ± 0.35	51.56 ± 0.38	0.9891
	UVB	0.1	0.1–3.00	0.1–2.5	−5.07 ± 0.68	45.39 ± 0.41	0.9339
	UVC	0.5	0.5–3.00	0.5–2.5	−1.26 ± 0.17	54.16 ± 0.19	0.8117
2	UVA	0.2	0.2–3.00	0.2–2.5	−7.21 ± 0.57	54.97 ± 0.66	0.9284
	UVB	0.1	0.1–3.00	0.1–2.5	−5.97 ± 0.69	50.45 ± 0.79	0.9142
	UVC	0.7	0.7–3.00	0.7–2.5	−2.13 ± 0.31	57.69 ± 0.31	0.8690
5	UVA	0.1	0.1–3.00	0.1–2.5	−11.58 ± 0.32	56.81 ± 0.15	0.9946
	UVB	0.1	0.1–3.00	0.1–2.5	−6.96 ± 0.68	53.51 ± 0.78	0.8943
	UVC	0.7	0.7–3.00	0.7–2.5	−1.39 ± 0.27	58.21 ± 0.31	0.7982

**Table 3:** The percent changes in the light reflectance at 530 nm for NBT–Pluronic F–127 samples irradiated with 1 J/cm^2^ during 0–168 h of storage.

UV Radiation Range	Concentration of NBT (g/dm^3^)	The Percent Changes in the Light Reflectance at 530 nm (%)				
		0 h	24 h	48 h	72 h	168 h
UVA	1	0	10	15	19	36
UVB		0	23	27	33	40
UVC		0	4	8	10	18
UVA	2	0	19	28	33	44
UVB		0	30	36	40	49
UVC		0	8	14	17	32
UVA	5	0	19	28	33	44
UVB		0	31	37	40	49
UVC		0	8	14	18	33

## Data Availability

Data are available upon request by contacting the corresponding authors.
